# MERCURY-3: a randomized comparison of netarsudil/latanoprost and bimatoprost/timolol in open-angle glaucoma and ocular hypertension

**DOI:** 10.1007/s00417-023-06192-0

**Published:** 2023-08-24

**Authors:** Ingeborg Stalmans, Kin Sheng Lim, Francesco Oddone, Marek Fichtl, Jose I. Belda, Anton Hommer, Guna Laganovska, Cédric Schweitzer, Bogomil Voykov, Tomasz Zarnowski, Gábor Holló

**Affiliations:** 1 Department of Ophthalmology, University Hospitals UZ Leuven, Louvain, Belgium; 2 Research Group of Ophthalmology, Department of Neurosciences, Catholic University KU Leuven, Louvain, Belgium; 3 KCL Frost Eye Research Department, St Thomas’ Hospital, London, UK; 4 IRCCS Fondazione Bietti, Rome, Italy; 5 Department of Ophthalmology, First Faculty of Medicine, Charles University and General University Hospital in Prague, Prague, Czech Republic; 6 Department of Ophthalmology for Children and Adults, Second Faculty of Medicine, Charles University and University Hospital Motol in Prague, Prague, Czech Republic; 7 Department of Ophthalmology, Hospital Universitario de Torrevieja, Alicante, Spain; 8 Visionker Eye Clinic, Torrevieja, Spain; 9 Private Office for Ophthalmology and Optometry, Albertgasse 39/10, 1080 Vienna, Austria; 10 Riga Stradins University, P.Stradins Clinical University Hospital, Riga, Latvia; 11 CHU Bordeaux, Department of Ophthalmology, 33000 Bordeaux, France; 12 Univ. Bordeaux, Inserm, Bordeaux Population Health Research Center, Team LEHA, UMR 1219, 33000 Bordeaux, France; 13 Centre for Ophthalmology, University Hospital Tuebingen, Tuebingen, Germany; 14 Department of Diagnostics and Microsurgery of Glaucoma, Medical University, Lublin, Poland; 15 Tutkimusz Ltd, Solymár, Hungary; 16 Eye Center, Prima Medica Health Centers, Budapest, Hungary

**Keywords:** Bimatoprost/timolol, Glaucoma, Intraocular pressure, Netarsudil/latanoprost, Rho-kinase inhibitor

## Abstract

**Purpose:**

To compare the efficacy and safety of the fixed-dose combination (FDC) of netarsudil 0.02%/latanoprost 0.005% ophthalmic solution (NET/LAT; Roclanda^®^) with bimatoprost 0.03%/timolol maleate 0.5% (BIM/TIM; Ganfort^®^) ophthalmic solution in the treatment of open-angle glaucoma (OAG) and ocular hypertension (OHT).

**Methods:**

MERCURY-3 was a 6-month prospective, double–masked, randomized, multicenter, active-controlled, parallel-group, non-inferiority study. Patients (≥ 18 years) with a diagnosis of OAG or OHT in both eyes that was insufficiently controlled with topical medication (IOP ≥ 17 mmHg in ≥ 1 eye and < 28 mmHg in both eyes) were included. Following washout, patients were randomized to once-daily NET/LAT or BIM/TIM for up to 6 months; efficacy was assessed at Week 2, Week 4, and Month 3; safety was evaluated for 6 months. Comparison of NET/LAT relative to BIM/TIM for mean IOP at 08:00, 10:00, and 16:00 h was assessed at Week 2, Week 6, and Month 3. Non-inferiority of NET/LAT to BIM/TIM was defined as a difference of ≤ 1.5 mmHg at all nine time points through Month 3 and ≤ 1.0 mmHg at five or more of nine time points through Month 3.

**Results:**

Overall, 430 patients were randomized (NET/LAT, *n* = 218; BIM/TIM, *n* = 212), and all received at least one dose of study medication. Efficacy analyses were performed at Month 3 on 388 patients (NET/LAT, *n* = 184; BIM/TIM, *n* = 204). NET/LAT demonstrated non-inferiority to BIM/TIM, with a between-treatment difference in IOP of ≤ 1.5 mmHg achieved at all time points and ≤ 1.0 mmHg at the majority of time points (six of nine) through Month 3. Mean diurnal IOP during the study ranged from 15.4 to 15.6 mmHg and 15.2 to 15.6 mmHg in the NET/LAT and BIM/TIM groups respectively, with no between-group statistically significant difference. No significant differences were observed in key secondary endpoints. No serious, treatment-related adverse events (AEs) were observed, and AEs were typically mild/moderate in severity. The most common treatment-related AEs were conjunctival hyperemia (NET/LAT, 30.7%; BIM/TIM, 9.0%) and cornea verticillata (NET/LAT, 11.0%; BIM/TIM, 0%).

**Conclusions:**

Once-daily NET/LAT was non-inferior to BIM/TIM in IOP reduction in OAG and OHT, with AEs consistent with previous findings. NET/LAT offers a compelling alternative FDC treatment option for OAG and OHT.
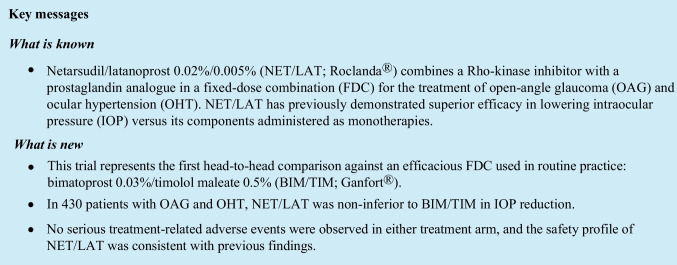

**Supplementary information:**

The online version contains supplementary material available at 10.1007/s00417-023-06192-0.

## Introduction

Controlling intraocular pressure (IOP), the key modifiable risk factor of conversion of ocular hypertension (OHT) to glaucoma and progression of open-angle glaucoma (OAG), remains the cornerstone of glaucoma management [1–4].

IOP is determined by aqueous humour (AH) production and AH drainage through trabecular (conventional) outflow to the episcleral veins, and uveoscleral (unconventional) outflow to the orbital venous system. Trabecular outflow is significantly influenced by the episcleral venous pressure (EVP) [5]. The trabecular pathway accounts for over 70% of AH outflow in the healthy young eye, which diminishes with advancing age as ocular rigidity increases [6]. Dysfunction of the trabecular meshwork (TM) contributes to increased resistance to AH outflow, driving an elevation in IOP [7, 8]. With the exception of pilocarpine and its secondary, mechanical, effect on the TM [9], topical glaucoma medications have primarily targeted AH production and/or uveoscleral outflow [4, 7, 8], and this approach has been largely unchanged over the past two decades.

Topical fixed-dose combination (FDC) formulations for lowering IOP have been increasingly used worldwide because they offer a convenient mode of administration that could potentially improve adherence, and may help to reduce toxicity related to cumulative preservative exposure if a patient is receiving more than one active topical ingredient [10]. Of the various FDC treatments available, combinations of a prostaglandin analogue (PGA) and timolol are commonly used outside the USA due to their strong IOP-lowering effect and once-daily instillation regimen [4, 11]. Despite current IOP management with FDC eyedrops, an estimated one-in-ten patients with OAG or OHT experience progression of glaucoma in the first 2.5 years following diagnosis [12], suggesting a need for additional treatment options.

Rho-kinase (ROCK) signalling has been identified in the TM and is an important regulator of trabecular outflow [13]. Inhibition of the ROCK signalling pathway has been associated with relaxation of the TM and with subsequent increase in AH outflow facility and reduction in IOP [14].

ROCK inhibitors are the first new class of drugs for glaucoma since the mid-1990s, and their unique mechanism of action represents an opportunity to evolve OAG/OHT treatment as a monotherapy or in combination with other drug classes with complementary mechanisms [15, 16]. Existing pre-clinical and clinical scientific evidence suggest that the ROCK inhibitor netarsudil lowers IOP through at least two mechanisms: by increasing trabecular outflow and by reducing EVP [5, 17–20]. Pivotal prospective investigations [21–23], and emerging real-world data, support the role of netarsudil in lowering IOP, both as monotherapy and in combination with latanoprost [19, 24].

The commercially available novel FDC of netarsudil and latanoprost, a commonly used PGA which increases uveoscleral outflow, has been developed to optimize complementary IOP-lowering mechanisms [22]. In previous randomized clinical trials, the FDC containing netarsudil and latanoprost has demonstrated a reduction in mean diurnal IOP over 3 months of 7.6–8.1 mmHg from baseline (mean 23.5–23.7 mmHg) in the management of OAG and OHT, with 58.4% of patients achieving an IOP ≤ 16.0 mmHg [21–23]. However, no head-to-head comparison of the IOP-lowering effect provided by the netarsudil/latanoprost FDC versus a PGA/timolol FDC has been performed to date.

Here, we present the first large-scale randomized prospective investigation of the efficacy and safety of netarsudil/latanoprost 0.02%/0.005% (NET/LAT; Roclanda^®^) ophthalmic solution in a head-to-head non-inferiority study against bimatoprost 0.03%/timolol maleate 0.5% (BIM/TIM; Ganfort^®^) ophthalmic solution. Of the available PGA/timolol FDCs, bimatoprost/timolol FDC was selected as the comparator in this investigation because of its highly efficacious PGA component [25, 26].

## Methods

### Study design and patients

MERCURY-3 (NCT03284853) was a 6-month prospective, double-masked, randomized, multicenter, active-controlled, parallel-group, non-inferiority study. Participants were evaluated at one of 58 clinical sites in 11 European countries (Supplementary Table 1). The study was initiated on 5 September 2017, with the last patient’s last visit on 6 November 2020.

The study was conducted in accordance with the Declaration of Helsinki, US FDA law, EU Clinical Trials Directive 2001/20/ED and in compliance with international guidelines (ICHGCP E6R2). Local and national regulatory requirements were followed as appropriate, and participants gave informed, written consent prior to entering the study.

Eligible participants were aged ≥ 18 years and had a diagnosis of OAG or OHT in both eyes that was insufficiently controlled by their existing treatment (medicated IOP ≥ 17 mmHg in at least one eye and < 28 mmHg in both eyes at screening). To qualify for the study, patients had to have been taking the same IOP-lowering medication for 30 days prior to screening. Between screening and the first treatment visit, a washout period was required (4 weeks for PGAs and β-adrenoceptor antagonists; 2 weeks for adrenergic agonists [including α-agonists such as brimonidine and apraclonidine]; and 5 days for muscarinic agonists [e.g., pilocarpine] and carbonic anhydrase inhibitors). Lid scrubs (which may have been used prior to, but not after, screening) and lubricating drops for dry eye (unrestricted use) were permitted.

The best-corrected visual acuity requirement was + 1.0 logarithm of the minimum angle resolvable (logMAR) or better by Early Treatment Diabetic Retinopathy Study (ETDRS) criteria in each eye (equivalent to 20/200 or better Snellen visual acuity in each eye). Before starting the masked study medication, the patients needed, as appropriate, a negative pregnancy test and/or willingness to use highly effective contraception during the study.

Treatment-naïve patients were excluded, as were those who were participating in any investigational study within 30 days prior to screening and those considered vulnerable (such as minors or adults under legal protection) or unable to express their consent (e.g., hospitalized persons in coma), persons deprived of liberty (prisoners from jails), or persons subject to psychiatric care. Ophthalmic exclusion criteria were as follows: clinically significant ocular disease; pseudoexfoliation or pigment dispersion glaucoma; a history of angle-closure glaucoma or eyes with complete or partial angle closure, and occludable anterior chamber angle (Shaffer grade < 2); previous laser peripheral iridotomy and/or an IOP ≥ 36.0 mmHg (unmedicated) in either eye. Medication-based exclusion criteria were: current (immediately prior to screening visit) treatment with BIM/TIM; use of more than two ocular hypotensive medications within 30 days of screening; and known hypersensitivity or contraindication to any component of the study medications or fluorescein. Any use of any topical steroid medication on the face or in, or around, the eyes was not permitted during the study, and use of steroid at screening was an exclusion criterion. Patients with prior insufficient IOP response/treatment failure (i.e., IOP did not reach the target range) with BIM/TIM ophthalmic solution were excluded. Glaucoma intraocular surgery (including laser treatments) as well as refractive surgery or ocular trauma in the six months prior to screening, and any other ocular surgery within three months prior to screening were also exclusion criteria.

Further exclusion criteria were a mean central corneal thickness > 620 µm at screening; any abnormality preventing reliable Goldmann applanation tonometry in either eye; recent or current evidence of ocular infection or inflammation, clinically significant blepharitis, conjunctivitis, herpes simplex, and/or keratitis. Patients were excluded if laboratory tests identified clinically significant abnormalities or previously unknown systemic disease deemed likely to interfere with the study. The use of any systemic medication, including corticosteroid use, with a possible effect on IOP was also an exclusion criterion.

### Endpoints

The primary objective of this study was to establish non-inferiority (a difference of ≤ 1.5 mmHg at all nine time points through Month 3 and ≤ 1.0 mmHg at five or more of nine time points through Month 3) for NET/LAT relative to BIM/TIM, and the primary efficacy outcome was a comparison of NET/LAT relative to BIM/TIM for mean IOP at 08:00, 10:00, and 16:00 h at Week 2, Week 6, and Month 3. Diurnal values were a mean of measurements taken during the day. Outcome comparisons of the study drugs were expressed as mean values and assessment of each time point was undertaken for the following secondary efficacy endpoints: diurnal IOP; change from diurnally adjusted baseline IOP at each study time point; change from baseline in mean diurnal IOP; percent change from diurnally adjusted baseline IOP; percentage change from baseline in mean diurnal IOP; and the percentage of patients achieving a pre-specified mean, mean change, and percentage mean change in diurnal IOP levels.

### Procedures

Once enrolled, participants were randomly assigned 1:1 to either NET/LAT or comparator BIM/TIM, both administered as one drop, once daily (QD) in both eyes in the evening between 20:00 and 22:00 h. Treatment continued with either NET/LAT or BIM/TIM for approximately 180 days (the safety endpoint). IOP measurements were taken at 08:00, 10:00, and 16:00 h at baseline and at Week 2 (Day 15), Week 6 (Day 43), and Month 3 (Day 90) using Goldmann applanation tonometry. One eye was termed the 'study' eye and the other the 'fellow eye'. The study eye was the eye with the higher IOP at 08:00 h on Visit 3; if both eyes had the same IOP at this visit, then the right eye was determined the study eye. Only the IOP values measured in the study eye were evaluated for efficacy. In each patient, both eyes received the study medication.

At each study visit, assessments were conducted for some or all of the following: systemic safety (heart rate, blood pressure and clinical laboratory evaluations, including hematology and clinical chemistry), pregnancy testing (for women of childbearing potential), ETDRS corrected visual acuity, objective findings of biomicroscopic evaluations (i.e., anterior segment evaluations including evaluation of cornea, conjunctiva, lids and lens), cup-to-disc ratio measurements, dilated ophthalmoscopic examination, quality of life assessments (NEI VFQ-25 and SF-36), and ocular symptoms and adverse events (AEs). Visual field testing was performed at screening, Month 3, and Month 6. For participants who discontinued treatment, every possible effort was made to conduct a final visit that included all study visit examinations at Month 6. Biomicroscopic grading of conjunctival hyperemia was performed on a standardized, 4-point scale: 0 = none (normal; appears white with a small number of conjunctival blood vessels easily observed); 1 = mild (prominent pinkish-red colour of both the bulbar and palpebral conjunctiva); 2 = moderate (bright, scarlet red colour of the bulbar and palpebral conjunctiva); 3 = severe (“beefy red” with petechiae; dark red bulbar and palpebral conjunctiva with evidence of subconjunctival hemorrhage) [27].

### Statistical methods

Two efficacy analysis sets were defined for statistical purposes, the intention-to-treat (ITT) population, which was used for all endpoints, and a per protocol (PP) set, which included participants without major protocol violations that were likely to seriously influence the primary outcome of the study as judged by a masked evaluation prior to the unmasking of the study treatment. Clinical non-inferiority for the experimental drug could be applied if the upper limit of the 95% confidence intervals (CI) around the difference was ≤ 1.5 mmHg at all nine time points through Month 3 and ≤ 1.0 mmHg at the majority of time points (at least five of nine) through Month 3. Each time point within each visit was modelled separately. Assuming no difference between NET/LAT and BIM/TIM, a two-tailed alpha of 0.05 (two-sided 95% CI) at each of the nine time points, a common SD of 3.5 mmHg, and a correlation between time points of 0.6 or less, 200 (ITT) participants per arm were necessary to have 85% power to show clinical non-inferiority (as defined above) for the primary endpoint. A randomization code for allocating the treatments was prepared by an independent biostatistician (Statistics & Data Corporation, Tempe, AZ and Waltham, MA, USA) who was not involved in the day-to-day conduct of the study. Study medication was provided in identical packaging to preserve masking.

To evaluate the primary outcome, a linear model was employed with IOP at the given visit and time point as the response, baseline IOP as a covariate, and treatment as a main effect factor at each time point at each visit. Markov Chain Monte Carlo (MCMC) multiple imputation techniques were used to impute missing data.

All patients who received at least one dose of treatment were included in the safety analysis population, and assessments took place until study completion or discontinuation.

## Results

### Participant disposition

**Fig. 1:**
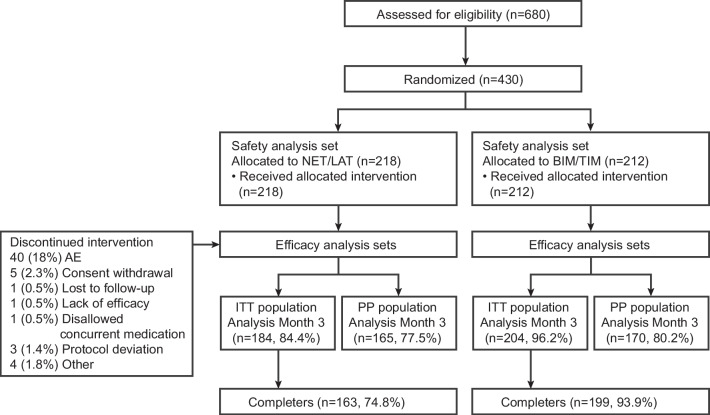
CONSORT diagram. AE, adverse event; BIM/TIM, bimatoprost 0.03%/timolol 0.5% FDC; FDC, fixed-dose combination; IOP, intraocular pressure; ITT, intention-to-treat; NET/LAT, netarsudil 0.02%/latanoprost 0.005% FDC; PP, per protocol

All of the 430 patients randomized (NET/LAT, *n* = 218; BIM/TIM, *n* = 212) received at least one dose of study medication and were included in the safety population, with efficacy analysis performed at Month 3 on 388 patients (NET/LAT, *n* = 184; BIM/TIM, *n* = 204; Fig. 1). Baseline characteristics were similar between study groups, although in the NET/LAT cohort there were more female patients and more participants who recorded prior use of PGAs compared with the BIM/TIM cohort (Table 1).

**Table 1:** Baseline characteristics

Characteristic *n* (%)	All participants *N* = 430		NET/LAT FDC *N* = 218		BIM/TIM FDC *N* = 212		*p* value
Age							0.7983
Mean, years (SD)	67.2	(11.65)	67.3	(12.03)	67.0	(11.27)	
Median (range)	69.0	(22, 91)	69.0	(25, 91)	68.5	(22, 91)	
≥ 65 years	280	(65.1)	147	(67.4)	133	(62.7)	0.3136
Sex, n (%)							
Female	223	(51.9)	131	(60.1)	92	(43.4)	0.0007**
Race, n (%)							
White	410	(95.3)	210	(96.3)	200	(94.3)	
Black/African American	9	(2.1)	4	(1.8)	5	(2.4)	0.2425
Asian	3	(0.7)		0	3	(1.4)	
Other/NA	8	(1.9)	4	(1.8)	4	(1.9)	
Iris colour of study eye, n (%)							
Brown/black	220	(51.2)	90	(41.3)	86	(40.6)	0.1203
Blue/grey/green	176	(40.9)	108	(49.5)	112	(52.8)	
Hazel	24	(5.6)	17	(7.8)	7	(3.3)	
Other	10	(2.3)	3	(1.4)	7	(3.3)	
Prior PGA hypotensive therapy, n (%)	318	(74.0)	171	(78.4)	147	(69.3)	0.0368*
Mean diurnal IOP mmHg in study eye at Day 1, mean (SD)	24.94	(3.33)	25.10	(3.41)	24.81	(3.26)	0.4560
Study eye diagnosis							
Open-angle glaucoma	236	(54.9)	124	(56.9)	112	(52.8)	0.4383
Ocular hypertension	194	(45.1)	94	(43.1)	100	(47.2)	

*p* values are from tests of differences across treatment groups and are two-sided. Fisher's exact tests were used for the categorical variables and one-way ANOVAs were used for the continuous variables

*p < 0.05; **p < 0.001

*ANOVA* analysis of variance; *BIM/TIM* bimatoprost 0.03%/timolol 0.5%; *FDC* fixed-dose combination; *NET/LAT* netarsudil 0.02%/latanoprost 0.005%; *SD* standard deviation

### Primary efficacy endpoint

**Fig. 2:**
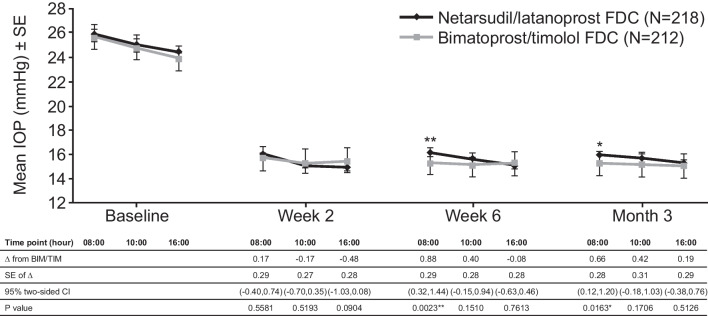
Ocular hypotensive efficacy assessed by mean IOP at Week 2, Week 6 and Month 3. *p < 0.05, **p < 0.01. Actual mean IOP values for comparison; analysis performed in ITT set. CI, confidence interval; IOP, intraocular pressure; ITT, intention-to-treat; SE, standard error

At the upper limit of the 95% CI difference, IOP ≤ 1.5 mmHg was achieved at all nine time points and ≤ 1.0 mmHg at the majority (six out of nine) of time points from Week 2 through Month 3 (Fig. 2). On the basis of these data, the primary endpoint of the study was met, and clinical non-inferiority of NET/LAT compared with BIM/TIM was demonstrated in the ITT population.

### Secondary efficacy endpoints

**Fig. 3:**
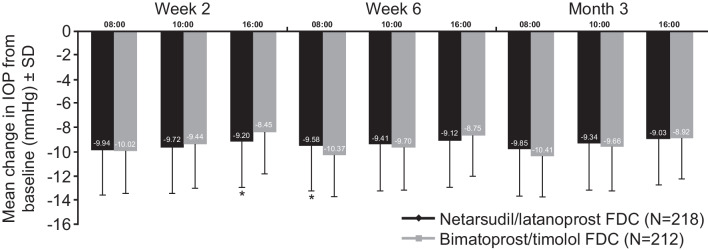
Actual mean IOP change at the pre-specified timepoints. *p < 0.005. Analysis performed in ITT set. FDC, fixed-dose combination; IOP, intraocular pressure; ITT, intention-to-treat; SD, standard deviation

**Fig. 4:**
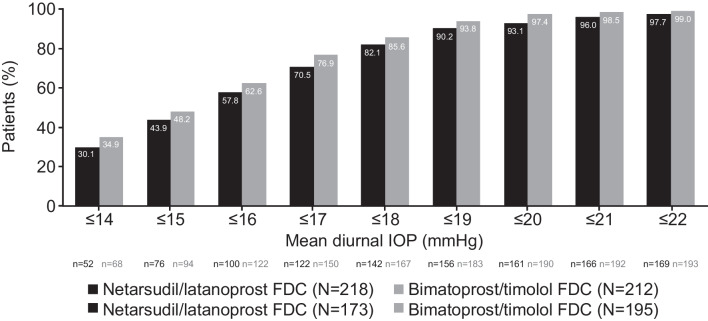
Percentage of patients reaching pre-specified categorical treatment targets at Month 3: mean diurnal IOP (mmHg). Analysis performed in ITT set. Baseline diurnal mean IOP: NET/LAT, 25.05 mmHg (SD ± 3.405); BIM/TIM, 24.81 mmHg (SD ± 3.256). N, number of patients randomized; n, number of patients with data included at this time point; FDC, fixed-dose combination; IOP, intraocular pressure; ITT, intention-to-treat

**Fig. 5:**
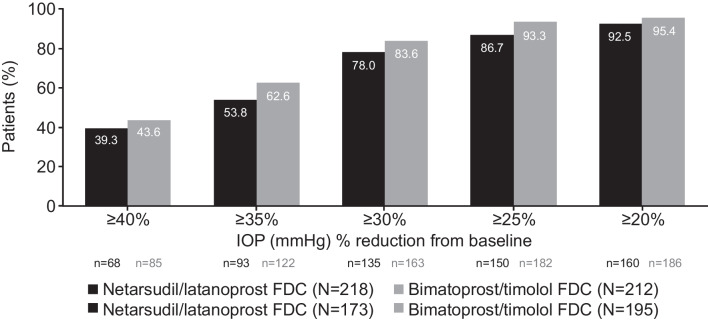
Percentage of patients reaching pre-specified categorical treatment targets at Month 3: percentage reduction from baseline in mean diurnal IOP. Analysis performed in ITT set. N, number of patients randomized; n, number of patients with data included at this time point. FDC, fixed-dose combination; IOP, intraocular pressure; ITT, intention-to-treat

The analysis for the primary efficacy endpoint was repeated on the PP population; the threshold for clinical non-inferiority was met at eight out of nine time points. Non-inferiority could not be confirmed overall for this population, because the upper bound 95% CI was 1.55 at the Week 6 08:00 time point. The mean IOP change at each of the pre-specified time points demonstrated clinical non-inferiority of NET/LAT relative to BIM/TIM using observed values (Fig. 3). No significant differences in mean change from diurnally adjusted baseline or diurnal IOP at Week 2, Week 6, and Month 3 were observed between the study drugs (Fig. 4). The number of patients achieving defined decrease from baseline in IOP at Month 3 was similar between treatment groups; 92.5% of the NET/LAT cohort and 95.4% of the BIM/TIM cohort achieved a clinically meaningful IOP reduction of ≥ 20% compared with baseline (Fig. 5).

The range of mean diurnal IOPs during the study period was 15.4 to 15.6 mmHg and 15.2 to 15.6 mmHg in the NET/LAT and BIM/TIM groups, respectively; no statistically significant differences were observed in the ITT analysis population. Mean change from diurnally adjusted baseline IOP at each study time point and at each study visit were calculated and no statistically significant differences were observed for the majority of endpoints. Mean diurnal IOP in the study eye at baseline was similar in both treatment groups: 25.1 mmHg for NET/LAT and 24.8 mmHg for BIM/TIM. Similar mean changes from diurnal baseline were observed at Month 3 for NET/LAT (– 9.4 mmHg) and BIM/TIM (–9.7 mmHg). No significant differences were observed in mean percent change in diurnal IOP from baseline for NET/LAT (–36.7%) and BIM/TIM (–38.6%) at Month 3 (*p* = 0.1056 [95% CI: –0.39, 4.05]).

Mean percent change from diurnally adjusted baseline IOP was similar for NET/LAT (–35.0% to –38.3%) compared to BIM/TIM (–34.8% to –40.2%) from Week 2 to Month 6; the majority of time points demonstrated no statistically significant difference between treatment groups. The efficacy profile for both treatment groups with or without prior PGA therapy is shown in Supplementary Fig. 1. Key secondary endpoints were consistent for the PP population (see Supplementary materials).

### Safety

The overall safety summary data are shown in Table 2. No serious treatment-related AEs were observed. Most AEs were ocular, and mild or moderate in severity. More treatment-emergent adverse events (TEAEs) were observed in the NET/LAT group; these were typically ocular with systemic TEAEs infrequent and evenly distributed between study groups. The overall discontinuation rate in the study due to TEAEs was 11.2%, with the majority of cases in the NET/LAT group (20.2% compared with 1.9% for BIM/TIM). Ocular AEs resulting in treatment discontinuation in ≥ 5% of patients observed in the NET/LAT group were as follows: conjunctival hyperemia (*n* = 14); allergic conjunctivitis (*n* = 9); cornea verticillata (*n* = 5); eye allergy (*n* = 4); foreign body sensation (*n* = 3), and conjunctival edema (*n* = 3). There was one death in the study (BIM/TIM arm); this was considered not related to treatment (traffic accident).

**Table 2:** Overall safety summary

	NET/LAT FDC (*N* = 218)	BIM/TIM FDC (*N* = 212)
TEAE, n	483	290
Ocular AE, n	352	131
Number of patients with ≥ 1 ocular AE, n (%)	131 (60.1)	64 (30.2)
Non–ocular AE, n	131	159
Number of patients with ≥ 1 non-ocular AE, n (%)	69 (31.7)	75 (35.4)
SAE, n	8	10
Number of patients with ≥ 1 SAE, n (%)	7 (3.2)	7 (3.3)
Treatment–related AE, n	291	91
Number of patients with ≥ 1 treatment-related AE, n (%)	120 (55.0)	53 (25.0)
Treatment–related SAE, n (%)	0	0
Number of patients with any AE, by maximum severity, n (%)		
Mild	64 (29.4)	65 (30.7)
Moderate	74 (33.9)	35 (16.5)
Severe	15 (6.9)	10 (4.7)

Analysis performed in the safety set

All AEs occurring during the study (i.e., once the subject had received one dose of study drug) were defined as TEAEs

Percentages are based on the number of subjects (N) in a given treatment group for the population being analyzed

*AE* adverse event; *BIM/TIM* bimatoprost 0.03%/timolol 0.5%; *FDC* fixed-dose combination; *NET/LAT* netarsudil 0.02%/latanoprost 0.005%; *SAE* serious adverse event; *TEAE* treatment-emergent adverse event

Treatment-related ocular AEs observed in ≥ 5% patients are summarised in Table 3; of these, conjunctival hyperemia and cornea verticillata were the most common, and all were more frequently observed in the NET/LAT group.

**Table 3:** Treatment-related ocular TEAEs

TEAE, n (%)	NET/LAT FDC (*N* = 218)	BIM/TIM FDC (*N* = 212)	*p* value
Conjunctival hyperemia	67 (30.7)	19 (9.0)	< 0.0001
Cornea verticillata	24 (11.0)	0	< 0.0001
Eye pruritus	17 (7.8)	2 (0.9)	< 0.0006
Punctate keratitis	12 (5.5)	4 (1.9)	0.0718
Conjunctivitis, allergic	11 (5.0)	1 (0.5)	0.0057

Analysis performed in the safety set

Percentages are based on the number of subjects (N) in a given treatment group for the population being analyzed

*BIM/TIM* bimatoprost 0.03%/timolol 0.5%; *FDC* fixed-dose combination; *NET/LAT* netarsudil 0.02%/latanoprost 0.005%; *TEAE* treatment-emergent adverse event

Through 6 months of study, the mean conjunctival hyperemia score was < 1 in both treatment groups (Supplementary Fig. 2).

Beyond the efficacy evaluation, change in IOP was collected as part of the safety assessment at 10:00 at Months 4, 5, and 6 (Supplementary Fig. 3) and was similar for both treatment groups. Diurnal mean IOP was similar in both treatment groups, which decreased to 15.6 mmHg (NET/LAT, *n* = 160) and 15.3 mmHg (BIM/TIM, *n* = 198). No significant differences were observed between treatment groups for slit lamp biomicroscopy findings. Ophthalmoscopy findings, cup–to–disc ratio, visual field, and vital signs (heart rate or blood pressure) were broadly similar with no notable differences between treatment groups. A statistically significant decrease in mean central corneal thickness was seen in the study and fellow eye from baseline to Month 3 (mean –6.2 µm, *p* < 0.001; –6.2 µm, *p* < 0.01, respectively), and Month 6 (mean –7.2 µm, *p* < 0.01; –7.5 µm, *p* < 0.05, respectively) for patients in the NET/LAT arm, which was not observed in the BIM/TIM group. Descriptive analyses found no meaningful impact of prior PGA therapy on occurrence of conjunctival hyperemia or discontinuation due to AEs (Supplementary Tables 2 and 3).

## Discussion

This 6-month prospective, double–masked, randomized, multicenter, active-controlled, parallel-group, non-inferiority study compared, for the first time, the efficacy and safety of NET/LAT and BIM/TIM in patients with OAG and OHT. For the primary efficacy analysis, NET/LAT FDC demonstrated non-inferiority to BIM/TIM, with a between-treatment difference in IOP of ≤ 1.5 mmHg achieved at all time points and ≤ 1.0 mmHg at the majority of time points (six of nine) from Week 2 through Month 3, the primary efficacy endpoint of the study.

The percentages of participants achieving a pre-specified mean, mean change, and percent mean change in diurnal IOP levels were similar in the two treatment groups throughout the study period. There were only two time points at which a statistically significant difference in mean IOP was observed: 08:00 at Week 6 and Month 3 in favour of BIM/TIM. A similar trend with regard to differences at specific time points has been observed in a previous work comparing once-daily netarsudil with twice-daily timolol [28]. Timolol is a beta-receptor blocker that accumulates in the ocular tissue and acts on sympathetic stimulation on waking [29, 30], which could influence IOP in the BIM/TIM arm at the early morning time point.

In clinical practice, the differences of < 1.0 mmHg (0.9 mmHg and 0.7 mmHg observed at 08:00 at Week 6 and Month 3) are unlikely to be clinically meaningful.

Treatment groups were broadly similar in baseline characteristics, although a between-group disparity was observed in prior use of PGA therapy. Despite more participants recording prior PGA use in the NET/LAT arm, and despite high intra-cohort variability in length of this PGA use, prior PGA exposure was not associated with meaningful differences in efficacy or treatment discontinuation.

Overall, no serious treatment-related AEs were observed, and AEs were mostly mild or moderate in severity in both treatment groups. The safety profile was consistent with previous MERCURY analyses [21–23], which evaluated NET/LAT against its components administered as monotherapy.

Although incidences of conjunctival hyperemia were significantly higher in the NET/LAT arm, the mean conjunctival hyperemia severity score was below 1 (mild) at all time points in both treatment arms and the majority of patients experiencing conjunctival hyperemia continued the study medication. Conjunctival hyperemia events reported in the NET/LAT arm (33.0%) were considerably lower than those reported in the earlier MERCURY-1 (53.4%) and MERCURY-2 (54.5%) studies conducted in the USA [22, 23]. The current results, along with increasing global clinical experience [24], suggest that NET/LAT is a suitable treatment option for most patients with OAG and OHT.

Conjunctival hyperemia in NET/LAT users can be explained by the vasodilatory effects of both components of the FDC, the ROCK inhibitor and the PGA. Latanoprost has previously been associated with an increased incidence of conjunctival hyperemia compared with timolol [31]. Additionally, it has been previously shown that the addition of timolol to a PGA reduces the associated conjunctival hyperemia [32]. While the mechanism driving this reduction is not yet fully understood, it is possible that, at least to some degree, the difference in conjunctival hyperemia between the treatment arms was due to timolol in the comparator arm.

Real-world experience with NET/LAT can provide context to the conjunctival hyperemia observations in the MERCURY trials. A recent study found that incidence of conjunctival hyperemia in routine practice was lower (32.9%) than in the randomized controlled clinical trials (53.4% to 63.0%) [24].

Cornea verticillata was only reported in the NET/LAT arm. This TEAE has been reported in the previous MERCURY Phase 3 studies where incidences were asymptomatic and had no impact on patient visual acuity [22, 23]. It is thought to be caused by a process of phospholipidosis, similar to that seen with the anti-arrhythmic drug amiodarone [33]. Consistent with previous studies, reports of cornea verticillata in MERCURY-3 were generally bilateral, mild in severity, and resolved or stabilized after stopping the study drug.

The observation of a reduced central corneal thickness in the NET/LAT arm was consistent with previous findings that suggest an additive effect of netarsudil and latanoprost, with the change (mean –6.4 µm in the MERCURY-2 *post hoc* analysis, mean –6.2 µm in MERCURY-3) unlikely to be clinically meaningful [34].

Despite the prospective, randomized, multicenter design of this study, it has limitations. The strict inclusion and exclusion criteria may have resulted in a study population that is not fully representative of real-world patients. As ever, controlled trial data should be complemented by clinic-based observational research. Stringent trial processes were challenging to follow for some investigators, resulting in a relatively high proportion of patients affected by protocol deviations. To account for the possibility that these deviations may have impacted on findings, a PP population was analyzed, and the findings were consistent with those of the ITT population; however, the PP group was not statistically powered to determine significance and so conclusions reached using this population must remain speculative. Because disease progression is common in treated patients, a longer-term study that could provide insight into the effects of NET/LAT on progression of glaucoma would be valuable and welcome.

In conclusion, the MERCURY studies show, overall, that NET/LAT offers a compelling, alternative FDC treatment option for OAG and OHT. The current MERCURY-3 study presents the first European NET/LAT study clinical data, derived from a large-scale, head-to-head comparison with BIM/TIM. By combining the ROCK inhibitor netarsudil (which increases conventional outflow by targeting TM dysfunction) with latanoprost (which increases unconventional outflow), this study demonstrates that NET/LAT FDC effectively lowers IOP to a similar degree to that of BIM/TIM.

### Supplementary information

Below is the link to the electronic supplementary material.

Supplementary file1 (DOCX 39.5 kb)

Supplementary Fig. 1 **Differences in IOP according to prior prostaglandin therapy status.** Descriptive analysis. Data from the ITT population. *FDC, fixed-dose combination; IOP, intraocular pressure; ITT, intention-to-treat; PGA, prostaglandin analogue; SE, standard error*. (PDF 552 kb)

Supplementary Fig. 2 **Mean conjunctival hyperemia score over 6 months.** Study eye conjunctival hyperemia score at 10:00 at each study visit in the safety population. Biomicroscopic grading of conjunctival hyperemia was performed on a standardized, 4-point scale: 0=none (normal; appears white with a small number of conjunctival blood vessels easily observed); 1 = mild (prominent pinkish-red color of both the bulbar and palpebral conjunctiva); 2=moderate (bright, scarlet red color of the bulbar and palpebral conjunctiva); 3 = severe (“beefy red” with petechiae; dark red bulbar and palpebral conjunctiva with evidence of subconjunctival hemorrhage).^1^ *FDC, fixed dose combination*. (PDF 433 kb)

Supplementary Fig. 3 **Change in IOP during the 6-month study period to assess significant changes in pressure.** Actual mean IOP at 10:00, collected as a safety measure. Observed data from the safety population. FDC, fixed-dose combination; IOP, intraocular pressure; SD, standard deviation. (PDF 474 kb)

Supplementary Table 1 **Distribution of sites by country and patient number.** A total of 68 sites were involved in the study, but patients were recruited from 58 sites only. (DOCX 34.5 kb)

Supplementary Table 2 **Patient incidence of hyperemia stratified by prior prostaglandin therapy.** Descriptive analysis, using treatment-emergent adverse event data from the intention-to-treat population. FDC, fixed-dose combination. (DOCX 34.3 KB)

Supplementary Table 3 **Patient discontinuation due to adverse events stratified by prior prostaglandin therapy.** Descriptive analysis. Data from the ITT population. AE, adverse event; FDC, fixed-dose combination; ITT, intention-to-treat. (DOCX 34.4 KB)
