# Supplementary material for: MERCURY-3: a randomized comparison of netarsudil/latanoprost and bimatoprost/timolol in open-angle glaucoma and ocular hypertension
Source: Graefes Arch Clin Exp Ophthalmol. 2023 Aug 24;262(1):179–90. doi: 10.1007/s00417-023-06192-0 (PMC10806046; doi:10.1007/s00417-023-06192-0)
Supplement: Supplementary file 1 — (DOCX 39.5 kb) [file 417_2023_6192_MOESM1_ESM.docx]

**Title:** MERCURY-3: a randomized comparison of netarsudil/latanoprost and bimatoprost/timolol in open-angle glaucoma and ocular hypertension

**Authors:**

Ingeborg Stalmans, MD, PhD,^1,2^ Kin Sheng Lim, MD,^3^ Francesco Oddone, MD,^4^ Marek Fichtl, MD,^5,6^ Jose I. Belda, MD, PhD,^7,8^ Anton Hommer, MD,^9^ Guna Laganovska, MD,^10^ Cédric Schweitzer, MD,^11,12^ Bogomil Voykov, MD, MBA,^13^ Tomasz Zarnowski, MD, PhD, DSc,^14^ Gábor Holló, MD, PhD, DSc^15,16^

**Affiliations:**

^1^Department of Ophthalmology, University Hospitals UZ Leuven, Leuven, Belgium; ^2^Research Group of Ophthalmology, Department of Neurosciences, Catholic University KU Leuven, Leuven, Belgium; ^3^KCL Frost Eye Research Department, St Thomas’ Hospital, London, UK; ^4^IRCCS Fondazione Bietti, Rome, Italy; ^5^Department of Ophthalmology, First Faculty of Medicine, Charles University and General University Hospital in Prague, Prague, Czech Republic; ^6^Department of Ophthalmology for Children and Adults, Second Faculty of Medicine, Charles University and University Hospital Motol in Prague, Prague, Czech Republic; ^7^Department of Ophthalmology, Hospital Universitario de Torrevieja, Alicante, Spain; ^8^Visionker Eye Clinic, Torrevieja, Spain; ^9^Private office for Ophthalmology and Optometry; Albertgasse 39/10; A-1080 Vienna; Austria; ^10^Riga Stradins University, P.Stradins Clinical University Hospital, Latvia; ^11^CHU Bordeaux, Department of Ophthalmology, F-33000 Bordeaux, France; ^12^Univ. Bordeaux, Inserm, Bordeaux Population Health Research Center, Team LEHA, UMR 1219, F-33000 Bordeaux, France; ^13^Centre for Ophthalmology, University Hospital Tuebingen, Tuebingen, Germany; ^14^Department of Diagnostics and Microsurgery of Glaucoma, Medical University, Lublin, Poland; ^15^Tutkimusz Ltd, Solymár, Hungary; ^16^Eye Center, Prima Medica Health Centers, Budapest, Hungary

**Corresponding author:**

Prof. Ingeborg Stalmans [ingeborg.stalmans@mac.com]

**Supplementary materials**

**Secondary analyses for the per protocol (PP) population**

No significant differences in mean change from diurnally adjusted baseline, or diurnal intraocular pressure (IOP), at Week 2, Week 6, and Month 3 were observed between study drugs. Mean diurnal IOP in the study eye at baseline was similar between both treatment groups, as was mean percent change from diurnally adjusted baseline IOP. For the percentages of participants achieving pre-specified mean, mean change, and percent mean change in diurnal IOP levels, no statistically significant differences were seen in diurnal mean IOP of ≤22, ≤21, ≤20, ≤19, ≤18, ≤17, ≤16, ≤15, ≤14 mmHg or in IOP reduction from baseline ≥2, ≥4, ≥6, ≥8, ≥10, ≥12 mmHg at Week 2, Week 6, or Month 3, or for IOP percent reduction from baseline of ≥5, ≥30, ≥35, ≥40 mmHg. These findings were consistent across the intention-to-treat (ITT) and PP groups.

**Safety analyses: key adverse events (AEs)**

The majority of conjunctival hyperemia cases were mild–to–moderate in severity, sporadic in nature for 78.7% of patients who experienced conjunctival hyperemia and completed the study, and ~80% of participants experiencing conjunctival hyperemia remained on study medication. In the netarsudil 0.02%/latanoprost 0.005% ophthalmic solution (NET/LAT; Roclanda^®^) group, 67 patients (30.7%) were affected by treatment-related cases of conjunctival hyperemia compared with 19 (9.0%) in the bimatoprost 0.03%/timolol maleate 0.5% (BIM/TIM; Ganfort^®^) group. The greatest percentage of clinically significant hyperemia in the NET/LAT arm, as assessed by the investigator, was seen at Month 3 with 16.7% (p<0.0001 vs BIM/TIM). For cornea verticillata, no patients in the BIM/TIM group experienced an event; the 24 cases (11.0%) in the NET/LAT group were all mild or moderate in severity.

The use of artificial tears was permitted at any time during the study period. Overall, 28.9% (n=63) of patients in the NET/LAT arm and 11.8% (n=25) of patients in the BIM/TIM arm received concomitant artificial tears during the study. Data on the duration, frequency, or date of onset of use were not collected. Of the 25 NET/LAT-treated patients who reported either eye pruritus or conjunctival allergy as a treatment-related AE, four received treatment with concomitant anti-allergy eyedrops.

Visual field examination data were collected at screening, Month 3, and Month 6; findings met requirements for automated threshold visual field assessment (e.g., 30–2 or 24–2 Humphrey or Octopus perimetry) and reliability. Baseline mean visual field mean deviations were similar in the NET/LAT and BIM/TIM groups. During the study, there were no notable differences in visual field results between the NET/LAT and BIM/TIM treatment groups. One treatment-emergent abnormal visual field test and two treatment-emergent cases of visual field defect were observed in the BIM/TIM group.

Treatment-related reductions in visual acuity were recorded for three patients in the NET/LAT group (two of which led to treatment discontinuation) and one in the BIM/TIM group. Mean visual acuity was relatively stable for all patients, with mean logMAR change from baseline of 0.005 (SD, 0.563) and 0.000 (SD, 0.0582) for NET/LAT and BIM/TIM, respectively. Post-hoc analysis found no meaningful impact of prior prostaglandin analogue (PGA) therapy on discontinuation due to AE or occurrence of conjunctival hyperemia.

**Supplementary Table 1: Distribution of sites by country and patient number**

A total of 68 sites were involved in the study, but patients were recruited from 58 sites only.

**Supplementary Table 2: Patient incidence of hyperemia stratified by prior PGA therapy**

Descriptive analysis, using treatment-emergent adverse event data from the ITT population.

FDC, fixed-dose combination; ITT: intention-to-treat; PGA, prostaglandin analogue.

**Supplementary Table 3: Patient discontinuation due to AEs stratified by prior PGA therapy**

Descriptive analysis. Data from the ITT population.

AE, adverse event; FDC, fixed-dose combination; ITT, intention-to-treat; PGA, prostaglandin analogue*.*

**Supplementary Fig. 1: Differences in IOP according to prior PGA therapy status**

Descriptive analysis. Data from the ITT population.

FDC, fixed-dose combination; IOP, intraocular pressure; ITT, intention-to-treat; PGA, prostaglandin analogue; SE, standard error.

**Supplementary Fig. 2: Mean conjunctival hyperemia score over 6 months**

Study eye conjunctival hyperemia score at 10:00 at each study visit in the safety population.
Biomicroscopic grading of conjunctival hyperemia was performed on a standardized, 4-point scale: 0=none (normal; appears white with a small number of conjunctival blood vessels easily observed); 1 = mild (prominent pinkish-red color of both the bulbar and palpebral conjunctiva); 2=moderate (bright, scarlet red color of the bulbar and palpebral conjunctiva); 3 = severe (“beefy red” with petechiae; dark red bulbar and palpebral conjunctiva with evidence of subconjunctival hemorrhage).^1^

FDC, fixed-dose combination.

**Supplementary Fig. 3: Change in IOP during the 6-month study period to assess significant changes in pressure**

Actual mean IOP at 10:00, collected as a safety measure. Observed data from the safety population.

FDC, fixed-dose combination; IOP, intraocular pressure; SD, standard deviation.
