# Supplementary figures and images for: MERCURY-3: a randomized comparison of netarsudil/latanoprost and bimatoprost/timolol in open-angle glaucoma and ocular hypertension
Source: Graefes Arch Clin Exp Ophthalmol. 2023 Aug 24;262(1):179–90. doi: 10.1007/s00417-023-06192-0 (PMC10806046; doi:10.1007/s00417-023-06192-0)

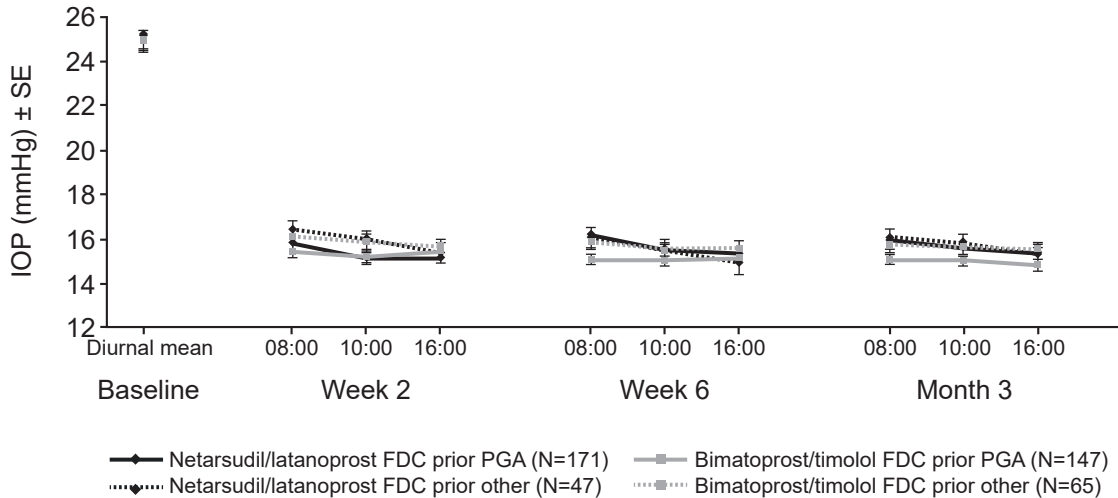

Supplement: Supplementary file 2 — Differences in IOP according to prior prostaglandin therapy status. Descriptive analysis. Data from the ITT population. FDC, fixed-dose combination; IOP, intraocular pressure; ITT, intention-to-treat; PGA, prostaglandin analogue; SE, standard error. (PDF 552 kb) [file 417_2023_6192_MOESM2_ESM.pdf]

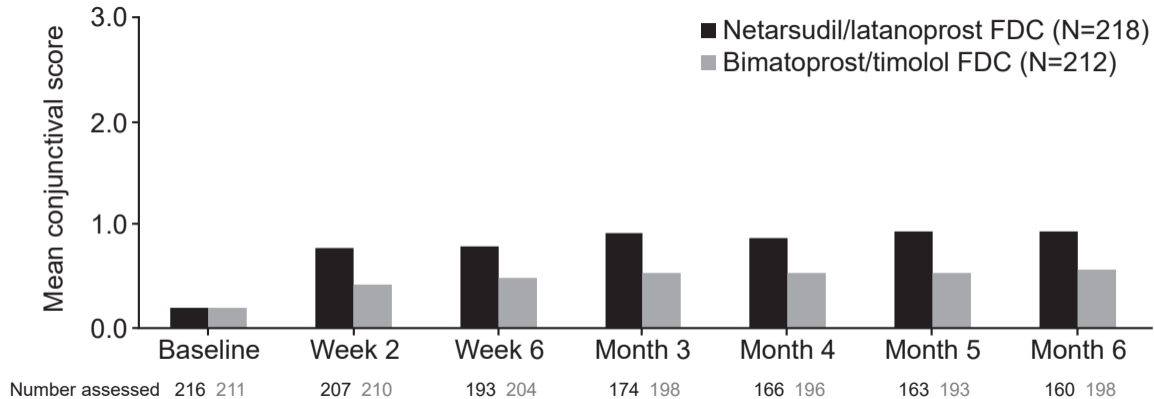

Supplement: Supplementary file 3 — Mean conjunctival hyperemia score over 6 months. Study eye conjunctival hyperemia score at 10:00 at each study visit in the safety population. Biomicroscopic grading of conjunctival hyperemia was performed on a standardized, 4-point scale: 0=none (normal; appears white with a small number of conjunctival blood vessels easily observed); 1 = mild (prominent pinkish-red color of both the bulbar and palpebral conjunctiva); 2=moderate (bright, scarlet red color of the bulbar and palpebral conjunctiva); 3 = severe (“beefy red” with petechiae; dark red bulbar and palpebral conjunctiva with evidence of subconjunctival hemorrhage).1 FDC, fixed dose combination. (PDF 433 kb) [file 417_2023_6192_MOESM3_ESM.pdf]

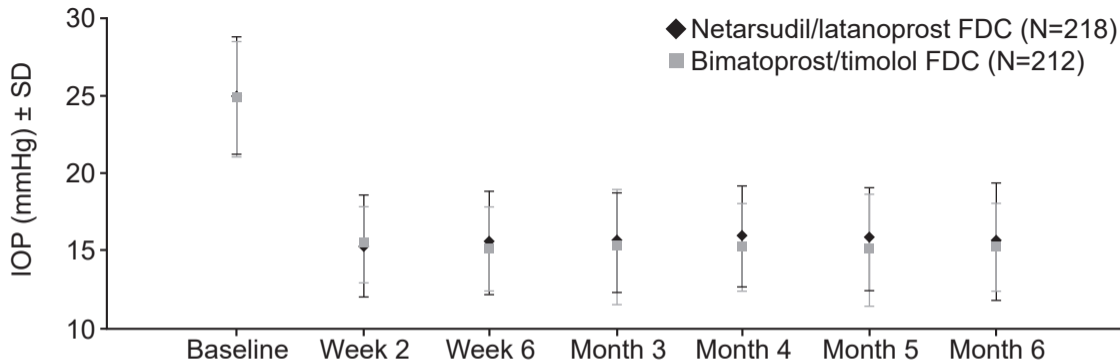

**Mean IOP mmHg**

|                            |       |       |       |       |       |       |       |
|----------------------------|-------|-------|-------|-------|-------|-------|-------|
| Netarsudil/latanoprost FDC | 24.99 | 15.31 | 15.52 | 15.56 | 15.89 | 15.78 | 15.61 |
| n                          | 218   | 208   | 194   | 175   | 166   | 163   | 160   |
| Bimatoprost/timolol FDC    | 24.84 | 15.41 | 15.15 | 15.22 | 15.20 | 15.06 | 15.23 |
| n                          | 212   | 211   | 206   | 198   | 196   | 193   | 198   |

Supplement: Supplementary file 4 — Change in IOP during the 6-month study period to assess significant changes in pressure. Actual mean IOP at 10:00, collected as a safety measure. Observed data from the safety population. FDC, fixed-dose combination; IOP, intraocular pressure; SD, standard deviation. (PDF 474 kb) [file 417_2023_6192_MOESM4_ESM.pdf]
