# Supplementary material for: MERCURY-3: a randomized comparison of netarsudil/latanoprost and bimatoprost/timolol in open-angle glaucoma and ocular hypertension
Source: Graefes Arch Clin Exp Ophthalmol. 2023 Aug 24;262(1):179–90. doi: 10.1007/s00417-023-06192-0 (PMC10806046; doi:10.1007/s00417-023-06192-0)
Supplement: Supplementary file 5 — Distribution of sites by country and patient number. A total of 68 sites were involved in the study, but patients were recruited from 58 sites only. (DOCX 34.5 kb) [file 417_2023_6192_MOESM5_ESM.docx]

| Country | Number of sites | Number of patients |
| --- | --- | --- |
| Austria | 3 | 34 |
| Belgium | 1 | 10 |
| Czech Republic | 2 | 61 |
| France | 3 | 6 |
| Germany | 4 | 74 |
| Hungary | 6 | 16 |
| Italy | 9 | 42 |
| Latvia | 3 | 19 |
| Poland | 3 | 6 |
| Spain | 13 | 127 |
| UK | 11 | 35 |
