# Supplementary material for: MERCURY-3: a randomized comparison of netarsudil/latanoprost and bimatoprost/timolol in open-angle glaucoma and ocular hypertension
Source: Graefes Arch Clin Exp Ophthalmol. 2023 Aug 24;262(1):179–90. doi: 10.1007/s00417-023-06192-0 (PMC10806046; doi:10.1007/s00417-023-06192-0)
Supplement: Supplementary file 6 — Patient incidence of hyperemia stratified by prior prostaglandin therapy. Descriptive analysis, using treatment-emergent adverse event data from the intention-to-treat population. FDC, fixed-dose combination. (DOCX 34.3 KB) [file 417_2023_6192_MOESM6_ESM.docx]

|  | **Netarsudil 0.02%/**  **latanoprost 0.005% FDC** | | | **Bimatoprost 0.03%/**  **timolol 0.5% FDC** | | |
| --- | --- | --- | --- | --- | --- | --- |
|  | **Total  N=218** | **Patients with prior PGA therapy**  n=171 | **Patients with no prior PGA therapy**  n=47 | **Total N=212** | **Patients with prior PGA therapy**  n=147 | **Patients with no prior PGA therapy**  n=65 |
| Hyperemia, n (%) | 72 (33) | 56 (78) | 16 (22) | 23 (11) | 17 (74) | 6 (26) |
| No hyperemia, n (%) | 146 (67) | 115 (79) | 31 (21) | 189 (89) | 130 (69) | 59 (31) |
