# Supplementary material for: MERCURY-3: a randomized comparison of netarsudil/latanoprost and bimatoprost/timolol in open-angle glaucoma and ocular hypertension
Source: Graefes Arch Clin Exp Ophthalmol. 2023 Aug 24;262(1):179–90. doi: 10.1007/s00417-023-06192-0 (PMC10806046; doi:10.1007/s00417-023-06192-0)
Supplement: Supplementary file 7 — Patient discontinuation due to adverse events stratified by prior prostaglandin therapy. Descriptive analysis. Data from the ITT population. AE, adverse event; FDC, fixed-dose combination; ITT, intention-to-treat. (DOCX 34.4 KB) [file 417_2023_6192_MOESM7_ESM.docx]

|  | **Netarsudil 0.02%/**  **latanoprost 0.005% FDC** | | | **Bimatoprost 0.03%/**  **timolol 0.5% FDC** | | |
| --- | --- | --- | --- | --- | --- | --- |
|  | **Total N=218** | **Patients with prior PGA therapy**  n=171 | **Patients with no prior PGA therapy**  n=47 | **Total N=212** | **Patients with prior PGA therapy**  n=147 | **Patients with no prior PGA therapy**  n=65 |
| Discontinuation due to AE, n (%) | 40 (18) | 28 (70) | 12 (30) | 4 (2) | 2 (50) | 2 (50) |
| Did not discontinue due to AE, n (%) | 178 (82) | 143 (80) | 35 (20) | 208 (98) | 145 (70) | 63 (30) |
